# AMA‐Negative Primary Biliary Cholangitis and Autoimmune Hepatitis Overlap Syndrome Presenting With Bullous Pemphigoid: A Case Report

**DOI:** 10.1002/ccr3.71534

**Published:** 2025-11-27

**Authors:** Tahmina Haque, Rafay Shahab Ansari, Ashiqur Rahman, Fahad Gul, Ashan Fareed, Sardar Noman Qayyum

**Affiliations:** ^1^ Comilla Medical College Cumilla Bangladesh; ^2^ College of Medicine Ziauddin University Karachi Pakistan; ^3^ Holy Family Red Crescent Medical College Hospital Dhaka Bangladesh; ^4^ Shifa Clinical Research Center, Shifa International Hospital Islamabad Pakistan; ^5^ Al‐Nafees Medical College, Isra University Islamabad Pakistan; ^6^ Bacha Khan Medical College Mardan Pakistan

**Keywords:** antimitochondrial antibody‐negative primary biliary cholangitis, autoimmune hepatitis, bullous pemphigoid, overlap syndrome

## Abstract

Multiple autoimmune syndrome involves the concurrent presentation of at least three separate autoimmune conditions in one individual. When patients present with both autoimmune hepatitis (AIH) and primary biliary cholangitis (PBC) simultaneously, it is referred to as AIH‐PBC overlap syndrome. About 1%–3% of PBC patients and 7% of AIH patients present with the overlap syndrome. A 45‐year‐old woman presented with jaundice for 1.5 years along with skin lesions on her legs, abdomen, and upper chest for 3 weeks. It was associated with generalized itching and weight loss. Physical examination revealed anemia, jaundice, enlarged liver and multiple crusted lesions representing bullous pemphigoid (BP). Investigations showed microscopic hypochromic anemia, positive ANA, and negative anti‐Smith and anti‐double‐stranded DNA antibodies. Imaging showed hepatomegaly and porta hepatitis lymphadenopathy. Congestive gastropathy was also observed on endoscopy, and there was no biliary tract dilatation on MRCP. There were chronic inflammation, portal tract expansion, necrosis, and cholestasis on liver biopsy. Despite treatment, the patient succumbed 1 month postadmission. This case report reflects the challenges associated with the management of overlap syndrome, including primary biliary cholangitis, autoimmune hepatitis, and BP. Rapid deterioration of the patient highlighted the need for early diagnosis and management of such patients.

**Key Clinical Message**

AMA‐negative primary biliary cholangitis with autoimmune hepatitis, complicated by bullous pemphigoid, presents significant diagnostic and therapeutic challenges. This case highlights the aggressive nature of autoimmune overlap syndromes, emphasizing the need for prompt recognition, multidisciplinary management, and close monitoring, as delayed intervention can lead to rapid deterioration and fatal outcomes.

## 1 Introduction

Multiple autoimmune syndrome (MAS) is defined as the coexistence of at least three distinct autoimmune diseases in a single patient. Autoimmune hepatitis (AIH) is a chronic progressive hepatic inflammation due to an unknown cause, resulting in permanent fibrosis. It is a rare disease with an incidence of 1–2 cases per 100,000 individuals annually [1]. Primary biliary cholangitis (PBC) is characterized by progressive destruction of intrahepatic bile ducts, resulting in cholestasis, cirrhosis, and portal hypertension. It predominantly affects middle‐aged females, affecting 14.6/100,000 individuals worldwide [2]. Bullous pemphigoid (BP) is also an autoimmune disorder with subepidermal blisters that usually affects elderly people [3]. When the characteristics of AIH and PBC overlap in a patient, fulfilling at least two of three Paris criteria for each disease [4], the patients are classified as having AIH‐PBC overlap syndrome [5]. Antinuclear antibodies (ANA), and antismooth muscle antibodies (ASMA) are characteristic of type 1 AIH, while anti‐liver kidney microsomal type 1 antibodies (anti‐LKM1) are characteristic of type 2 AIH. In 95% of the PBC cases, antimitochondrial antibodies (AMA) are detected, and 5% of PBC cases are AMA‐negative [2]. The simultaneous presentation of seronegative PBC and AIH remains elusive, since nearly 95% of the PBC cases are tested positive for AMA. In the minority of cases lacking this marker, clinicians must rely on histopathology, atypical autoantibodies including anti‐gp 210 and anti‐Sp100, and a high index of suspicion to establish the diagnosis [4].

In this manuscript, we report a case of seronegative PBC, and type 1 AIH, the overlap syndrome, in a patient presenting with BP. This case has not been reported before and will add a valuable contribution to the growing literature.

## 2 Case Summary and Examination

**FIGURE 1:**
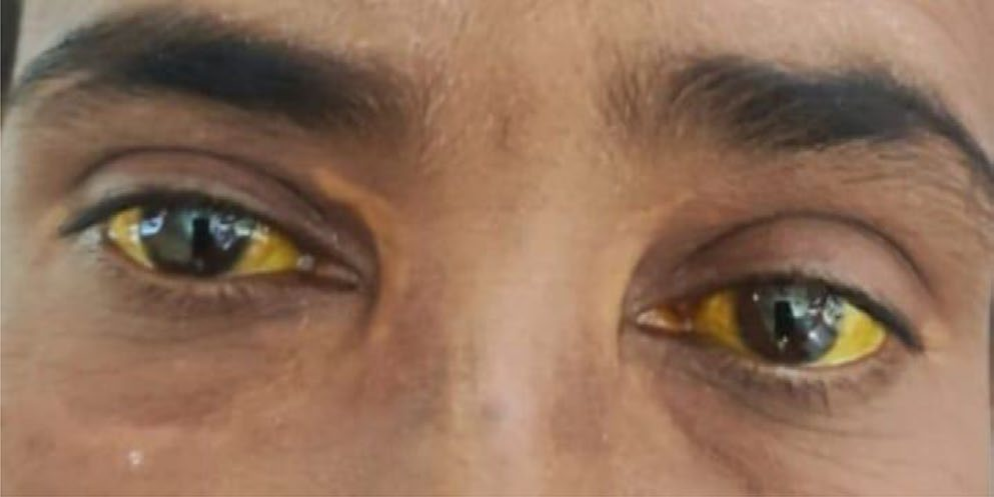
Deeply icteric scleral discoloration, indicative of jaundice associated with hepatic dysfunction from PBC and AIH.

A 45‐year‐old woman was admitted to the hospital with persisting jaundice for 1.5 years, and multiple tense, blister‐like skin lesions of varying morphology over the last 3 weeks. These lesions were more prominent on her upper chest, abdomen, and inner aspect of her left leg. Initially, the lesions contained clear fluid, then progressed to become pustular, and eventually healed by crusting. Additionally, the patient also experienced generalized itching across her body, which was constant throughout the day and night, particularly intense in her limbs. She also reported feeling fatigued, and noted that her clothes felt looser, suggesting possible weight loss. During the general physical examination, she appeared moderately anemic, and deeply icteric, as shown in Figure 1.

**FIGURE 2:**
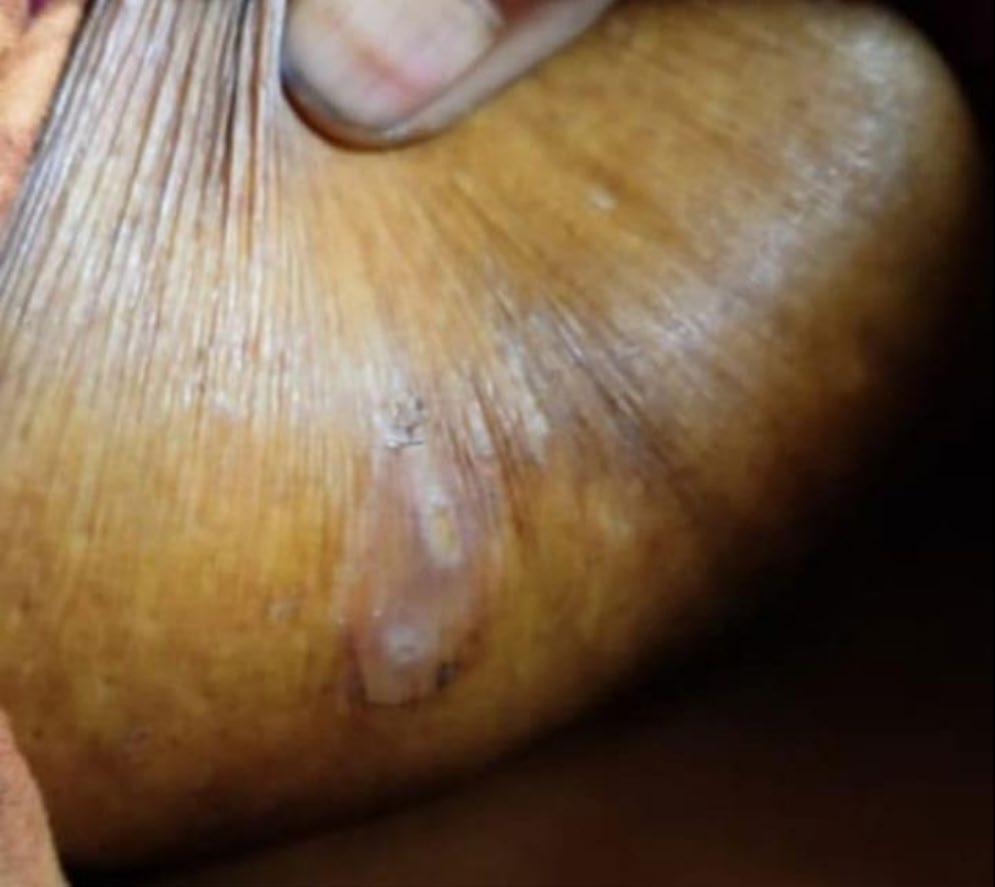
Crusted lesion in the mammary fold, consistent with bullous pemphigoid, showing signs of chronic inflammation and pruritus.

Multiple crusted skin lesions were also observed on her upper chest, mammary fold, abdomen, and inner aspects of her left leg, consistent with bullous pemphigoid, as shown in Figure 2.

Additionally, multiple xanthelasma were present on her face. Her heart rate was 66/min, and her blood pressure was 100/60 mmHg. During the abdominal examination, her liver was enlarged 3 cm below the right costal margin in the mid‐clavicular line. It was firm, and nontender with a smooth surface, and regular margins. Dullness was present at the upper border of the liver at the fifth intercostal space. Other systemic examination was unremarkable.

## 3 Investigations and Diagnosis

Microcytic hypochromic anemia was noted on peripheral blood film (Table 1), with a negative Coombs test. Antinuclear antibodies detected by indirect immunofluorescence tested positive. However, neither anti‐Smith antibodies, nor anti‐dsDNA antibodies were detected. Liver function tests revealed elevated levels of alkaline phosphate (ALP), aspartate aminotransferase (AST), and serum bilirubin, as demonstrated in Table 2.

**TABLE 1:** Hematological tests.

Laboratory tests	Patient's value	References
Hemoglobin	7.5 g/dL	13–17 g/dL
HCT	22.4%	40%–52%
WBC	17,000/mm^3^	4,000–11,000/mm^3^
Serum iron	42 μg/d149 to 492 mcg/dL	50 to 170 mcg/dL
Serum ferritin	1,132 ng/mL	13 to 150 ng/mL
TIBC	304 μg/dL	149 to 492 mcg/dL

**TABLE 2:** Liver function test.

Laboratory tests	Patient's value	References value
ALP	1540 U/L	46–116 U/L
ALT	291 U/L	10–49 U/L
Serum total bilirubin	33.1 mg/dL	0.2–1.2 mg/dL
Serum direct bilirubin	22.5 mg/dL	0.1–0.3 mg/dL
Serum indirect bilirubin	10.6 mg/dL	0.2–0.8 mg/dL
Prothrombin time	16 s	10–12 s
Bleeding time	03 min 45 s	1–5 s
Clotting time	05 min 10 s	Up to 11 s
HBsAg	Negative	
Anti‐HCV antibody	Negative	
Anti mitochondrial antibody	0.7 U/mL	< 0.95 = Negative 0.95–1 = Borderline > 1 = Positive
Antismooth muscle antibody	Titer—1:40 (negative)	
Liver‐kidney microsomal Antibody	Titer—1:40 (negative)	

Ultrasonography of the entire abdomen indicated mild hepatomegaly, and a contracted gallbladder, with suspected lymphadenopathy at the portal hepatis. Upper gastrointestinal endoscopy revealed congestive gastropathy, while colonoscopy was unremarkable. Magnetic resonance cholangiopancreatography (MRCP) confirmed mild hepatomegaly, and a contracted gallbladder, with no dilation of the biliary tracts.

**FIGURE 3:**
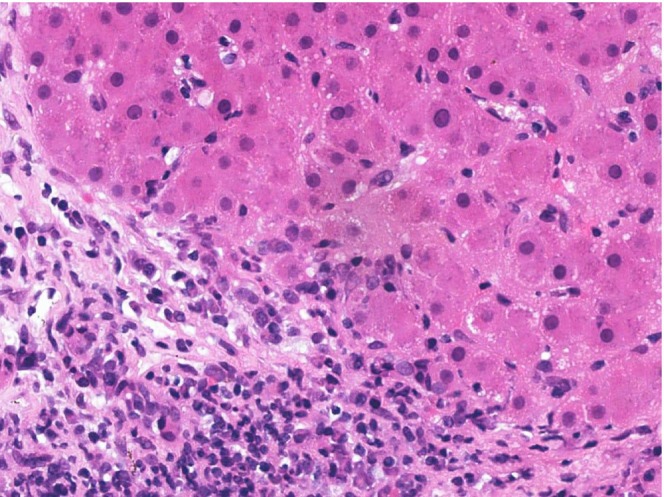
Moderate portal lymphoplasmacytic infiltrate with interface hepatitis (H&E × 40).

**FIGURE 4:**
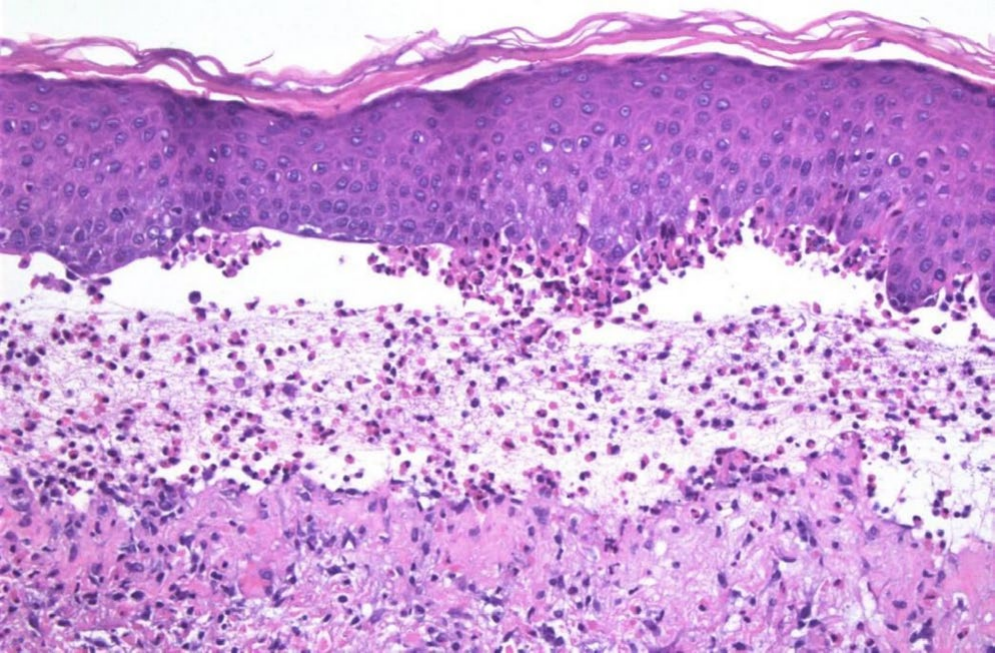
Subepidermal blister cavity showing numerous inflammatory cells, including eosinophils, characteristic of bullous pemphigoid (H&E × 40).

A liver biopsy was conducted, as shown in Figure 3, revealing moderate infiltration of chronic inflammatory cells and mild piecemeal necrosis in the portal area, with few eosinophils present. Karyorrhectic bodies were also observed. These findings were consistent with autoimmune hepatitis. Another section of the core biopsy revealed focal ballooning of hepatocytes, and signs of cholestasis. Additionally, there was portal tract expansion with a large number of inflammatory cells, predominantly lymphocytes, indicative of PBC. The diagnosis of AIH overlapping with PBC was made. Additionally, the biopsy of skin lesions also confirmed the diagnosis of bullous pemphigoid, as shown in Figure 4, which is also an autoimmune disease. The patient was diagnosed as a case of “multiple autoimmune syndrome.”

Table 3 shows the diagnostic workup of the patient, and Table 4 includes the differential diagnoses of the patient.

**TABLE 3:** Diagnostic Summary of the AIH‐PBC Overlap with Bullous Pemphigoid.

Step	Findings (as reported)	Interpretation/Contribution to diagnosis
Clinical presentation	Jaundice for 1.5 years, fatigue, weight loss, pruritus, and bullous skin lesions (legs, chest, abdomen)	Suggests chronic liver dysfunction and dermatological autoimmunity
General examination	Moderate anemia, deep jaundice, xanthelasma, crusted lesions consistent with BP	Supports systemic autoimmune pathology
Liver function tests	Elevated ALP, AST, total bilirubin	Mixed hepatocellular and cholestatic pattern
Hematology	Microcytic hypochromic anemia; Coombs test negative	Suggests chronic disease‐related anemia
Autoimmune markers	ANA: strongly positive; anti‐Smith & anti‐dsDNA: negative; AMA: negative	Suggests AIH; seronegative PBC suspected
Imaging studies	USG: Mild hepatomegaly, contracted gallbladder, porta hepatis lymphadenopathy MRCP: No biliary dilation	No extrahepatic obstruction; compatible with intrahepatic cholangiopathy
Endoscopy findings	Congestive gastropathy (upper GI)	Suggests portal hypertension component
Liver biopsy	Interface hepatitis with piecemeal necrosis, cholestasis, ductal injury, and lymphocyte infiltration	Diagnostic criteria for AIH‐PBC overlap syndrome
Skin biopsy + DIF	Subepidermal blister with eosinophils Linear IgG and C3 at the basement membrane	Diagnosis of bullous pemphigoid
Final diagnosis	AIH–PBC overlap syndrome (AMA‐negative) with suspected bullous pemphigoid	Multiple autoimmune syndrome confirmed based on the criteria

**TABLE 4:** Differential diagnoses and final diagnosis of AIH‐PBC Overlap Syndrome.

Differential diagnosis	Rationale for consideration	How it was ruled out
Drug‐induced liver injury	Cholestatic pattern common with statins, antibiotics	No relevant drug exposure; RUCAM score low
Viral hepatitis (A, B, C, E)	Jaundice + elevated transaminases	Negative viral serologies
Primary sclerosing cholangitis (PSC)	Cholestatic enzymes, cholangiopathy on imaging	MRCP showed no bile‐duct strictures; ANCA neg
Other immunobullous diseases (e.g., PV)	Blisters and pruritus could mimic BP	DIF pattern (linear IgG/C3) is diagnostic for BP
Secondary biliary cirrhosis	Chronic biliary obstruction	MRCP: no extrahepatic duct dilation
Overlap syndrome (AIH + PBC)	Mixed biochemical, serological, and histological features	Met Paris criteria; biopsy confirmed interface hepatitis + duct lesions

## 4 Treatment and Follow‐Up

The patient was commenced on ursodeoxycholic acid (UDCA) (15 mg/kg/day), prednisolone (40 mg/day) for presumed autoimmune hepatitis, and cholestyramine (4 g twice a day (BID)) for pruritus. Atorvastatin (10 mg/day) was introduced later for hyperlipidemia. Given the overlap syndrome, immunosuppression was considered but azathioprine was withheld due to baseline cytopenia. Unfortunately, the patient deteriorated and expired 1 month after admission.

## 5 Discussion

In this manuscript, we report a case of overlap syndrome of AIH and PBC, complicated by bullous pemphigoid (BP). The patient exhibited symptoms and laboratory findings characteristic of both PBC and AIH, such as cholestasis (impaired bile flow), elevated liver enzymes, positive ANA, and histological features in addition to bullous pemphigoid. About 10% of patients with AIH and PBC present with the AIH‐PBC overlap syndrome [6]. The patient's fatal outcome within 1 month of diagnosis underscores both the diagnostic challenges, and the aggressive nature of overlap syndromes, especially in the AMA‐negative cases, where standard serological markers are absent.

PBC is marked by the presence of chronic, progressive destruction of small intrahepatic bile ducts, typically in middle‐aged women, and is identified in 90%–95% of the cases by the presence of AMA against E2 subunit of pyruvate dehydrogenase (PDC‐E2) [7]. However, 5%–8% of the PBC patients lack detectable AMA by immunofluorescence or immunoblotting, termed as AMA‐negative PBC [8]. AMA‐negative patients often present later in the disease course, with more severe bile duct loss and higher rates of concomitant autoimmune conditions, reflecting a more aggressive phenotype [9]. AMA‐negative PBC shows the same nonsuppurative destructive cholangitis, and interlobular bile duct paucity seen in AMA‐positive cases, making liver biopsy impertinent for diagnosis in seronegative patients [10]. As in our patient, elevated ALP and cholestatic histology in the absence of AMA should prompt clinicians to pursue biopsy, and additional autoantibody panels, including ANA, ASMA, anti‐gp210, and anti‐sp100 [11].

Overlap syndromes occur when characteristics of two autoimmune liver diseases coexist. AIH‐PBC is the most frequently recognized overlap syndrome, accounting for about 10% of PBC and around 7% of AIH cases [2, 12]. The Paris criteria facilitate diagnosis by requiring at least two features from each disease across three domains: biochemical (ALP ≥ 2× upper limit of normal for PBC; ALT ≥ 5× upper limit of normal for AIH), serological (AMA‐positivity for PBC; ANA/ASMA for AIH), and histological (florid duct lesion for PBC; interface hepatitis for AIH) [12]. In AMA‐negative patients, the diagnosis is based on serology (ANA/ASMA titers) and histopathology, as demonstrated in our case, where strong ANA positivity and classic portal interface activity confirmed the diagnosis of overlap syndrome [13]. The patients meeting Paris criteria often exhibit a more severe form of the disease, faster progression to cirrhosis, and require combination therapy rather than UDCA monotherapy [14].

Bullous pemphigoid, an IgG‐mediated subepidermal blistering disorder targeting BP180, and BP230 antigens in hemidesmosomes, primarily affects the elderly, and may complicate autoimmune liver disease [15]. The coexistence of BP, with PBC was first reported in 1983, and remains exceedingly rare, with fewer than 10 cases reported to date [16, 17]. BP often arises concurrently or shortly following hepatic disease onset, indicating shared immunopathogenic pathways rather than mere coincidence [17]. Similarly in our patient, rapid progression of BP, refractory itching, and extensive blistering highlight the potential severity of the autoimmunity against hepatic and cutaneous structures. The underlying mechanism may involve epitope spreading, in which chronic bile duct injury exposes neoantigens, triggering cutaneous autoimmunity, or shared HLA susceptibility loci that predispose to both conditions [18].

Both autoimmune liver diseases and bullous pemphigoid share common immunogenetic and cellular features. AIH is found to be associated with HLA‐DR3 and DR4 in white populations, whereas PBC is linked to HLA‐DR8 and DQ loci. Therefore, overlap patients often carry multiple at‐risk alleles including HLA‐B8, DR3, and DR4 [19] [20]. These class II HLA molecules present self‐antigens, such as PDC‐E2, or BP180 peptides, to autoreactive CD4+ T cells, precipitating a maladaptive immune response. B‐cell hyperactivity, driven by T‐cell help, results in high titers of autoantibodies including AMA, ANA, ASMA, and anti‐BP180 that mediate tissue damage via complement activation, and recruitment of neutrophils, and eosinophils [11]. Infections, such as *E. coli* urinary tract infections in PBC, or drugs may initiate molecular mimicry and breach tolerance in generically susceptible individuals. In AMA‐negative PBC, alternate autoantigens, including gp210, and Sp100, may play a pathogenic role, and serve as diagnostic markers, particularly in seronegative patients with prominent portal inflammation [2, 9]. The convergence of these processes is the most probable cause of multisystem autoimmunity seen in multiple autoimmune syndromes (MAS). Our patient's triad constitutes a novel variant of MAS including AIH‐PBC overlap syndrome along with PB.

The management of AIH‐PBC overlap requires a tailored, often aggressive approach. First‐line therapy for PBC is UDCA, which improves cholestasis, delays histological progression, and enhances transplant‐free survival. However, only 25% of the AMA‐negative PBC patients attain a biochemical response to UDCA alone. AIH responds to corticosteroids such as prednisolone 0.5–1 g/kg/day and azathioprine, which reduce transaminases, IgG levels, and interface hepatitis [12]. In overlap syndrome, especially in the AMA‐negative cases, combining UDCA with immunosuppression is recommended to address both cholestatic and hepatitis components [12]. Bullous pemphigoid typically requires systemic steroids or immunomodulators such as mycophenolate mofetil and rituximab, but poses the challenges of potentially worsening hepatic inflammation and infection risks [15].

Despite the optimal therapy, AIH‐PBC overlap carries a poorer prognosis than isolated cases, with higher rates of cirrhosis, portal hypertension, and liver‐related mortality [21]. In addition, BP may further worsen the outcomes due to treatment‐related complications, sepsis, and systemic inflammation, as seen in similar cohorts where 1‐year survival dropped below 50% in overlap patients with dermatological involvement [17]. Our patient's rapid decline highlights the need for timely diagnosis, multidisciplinary care, and consideration of second‐line agents such as obeticholic acids (OCA) or biologics in refractory cases.

Although the patient expired after 1 month, this case highlights the need for clinicians to suspect seronegative PBC in patients with cholestatic enzymes but negative AMA; liver biopsy and additional antibodies testing are diagnostic. It also teaches clinicians to apply overlap criteria (Paris criteria) rigorously to identify AIH‐PBC early, even when enzyme patterns are mixed, which could help improve the prognosis. This case also highlights the need for prompt systemic evaluation for underlying AIH or PBC in cases of new‐onset bullous pemphigoid in a patient with liver dysfunction. The case also highlights that rapid progression in overlap syndromes requires early multidisciplinary team action, including hepatologists, dermatologists, and immunologists' collaborations.

Although the patient unfortunately succumbed rapidly, the retrospective analysis suggests several opportunities that might have altered her course: early and more aggressive immunosuppression with corticosteroids plus azathioprine could blunt interface hepatitis, and limit ductal injury. Second‐line therapies such as rituximab might have controlled hepatic autoimmunity and skin blistering with early administration. The use of adjunctive plasmapheresis in fulminant bullous pemphigoid could rapidly remove circulating anti‐BP 180/230 IgG, reducing blister formation.

## 6 Conclusion

This case report reflects the challenges associated with the management of overlap syndrome, including PBC, autoimmune hepatitis, and bullous pemphigoid. The accelerated disease progression observed in our patient emphasizes the necessity for prompt identification and intensive therapeutic intervention. Our findings add a valuable contribution to the existing literature and underscore the need for further research to develop diagnostic and therapeutic strategies to improve patient survival in these challenging scenarios.

## Author Contributions

**Tahmina Haque:** conceptualization, data curation, investigation, resources, supervision, writing – original draft, writing – review and editing. **Rafay Shahab Ansari:** software, supervision, writing – original draft. **Ashiqur Rahman:** conceptualization, writing – original draft, writing – review and editing. **Fahad Gul:** supervision, visualization, writing – original draft, writing – review and editing. **Ashan Fareed:** writing – original draft. **Sardar Noman Qayyum:** investigation, writing – original draft, writing – review and editing.

## Consent

As the patient died, consent to publish the case was taken from the patient's family. A signed copy of the consent form is available from the corresponding author. Upon reasonable request, it can be provided.

## Conflicts of Interest

The authors declare no conflicts of interest.

## Data Availability

The data that support the findings of this study are available on request from the corresponding author. The data are not publicly available due to privacy or ethical restrictions.
